# Imaging of anorectal malformations: where are we now? Abdominal imaging task force of the European Society of Paediatric Radiology

**DOI:** 10.1007/s00247-022-05395-7

**Published:** 2022-06-01

**Authors:** Samuel Stafrace, Luisa Lobo, Thomas A. Augdal, Fred Efraim Avni, Costanza Bruno, Maria Beatrice Damasio, Kassa Darge, Stéphanie Franchi-Abella, Jochen Herrmann, Donald Ibe, Damjana Kljucevsek, Hans-Joachim Mentzel, Marcello Napolitano, Aikaterini Ntoulia, Lil-Sofie Ording-Müller, Giulia Perucca, Philippe Petit, Anne M. Smets, Seema Toso, Magdalena Maria Woźniak, Michael Riccabona

**Affiliations:** 1 Department of Diagnostic Imaging, Sidra Medicine, Education City, Al Luqta Street, Doha, Qatar; 2 Weill Cornell Medicine, Doha, Qatar; 3 Serviço de Imagiologia Geral, Hospital de Santa Maria - Centro Hospitalar Universitário Lisboa, Norte (CHULN), Lisbon, Portugal; 4 Section for Paediatric Radiology, Department of Radiology, University Hospital of North Norway, Tromsø, Norway; 5 Department of Pediatric Radiology, Jeanne de Flandre Hospital, CHRU de Lille, Lille Cedex, France; 6 Department of Radiology, Azienda Ospedaliera Universitaria Integrata Verona (AOUI), Verona, Italy; 7 Radiology Department, IRCCS Istituto Giannina Gaslini, Genoa, Italy; 8 Department of Radiology, The Children’s Hospital of Philadelphia, University of Pennsylvania, Philadelphia, PA USA; 9 Paediatric Radiology Department, Paris-Saclay University, AP-HP, Hôpital Bicêtre, BIOMAPS UMR, 9011 Le Kremlin-Bicêtre, France; 10 Section of Pediatric Radiology, Department of Diagnostic and Interventional Radiology and Nuclear Medicine, University Medical Center Hamburg-Eppendorf, Martinistrasse 52, 20251 Hamburg, Germany; 11 Department of Radiology, Silhouette Diagnostic Consultants, Abuja, Nigeria; 12 Department of Radiology, University Children’s Hospital Ljubljana, Ljubljana, Slovenia; 13 Section of Pediatric Radiology, Institute of Diagnostic and Interventional Radiology, University Hospital, Jena, Germany; 14 Department of Paediatric Radiology and Neuroradiology, V. Buzzi Children’s Hospital, Milan, Italy; 15 Royal Belfast Hospital for Sick Children, Belfast, UK; 16 Division of Radiology and Nuclear Medicine, Department of Paediatric Radiology, Oslo University Hospital, Rikshospitalet, Oslo, Norway; 17 Department of Radiology, Great Ormond Street Hospital for Children NHS Foundation Trust, London, UK; 18 Service d’Imagerie Pédiatrique et Prénatale, Hôpital Timone Enfants, Aix Marseille Université, Marseille, France; 19 Department of Radiology and Nuclear Medicine, Amsterdam University Medical Centers, Amsterdam, The Netherlands; 20 Department of Pediatric Radiology, University Hospital of Geneva, Geneva, Switzerland; 21 Department of Pediatric Radiology, Medical University of Lublin, Lublin, Poland; 22 Department of Radiology, Division of Pediatric Radiology, Medical University Graz and University Hospital LKH, Graz, Austria

**Keywords:** Anorectal malformation, Anus, Children, Cloacal malformation, Fluoroscopy, Infants, Magnetic resonance imaging, Radiography, Rectum, Ultrasound

## Abstract

Anorectal and cloacal malformations are a broad mix of congenital abnormalities related to the distal rectum and anus. Confusion exists between all the forms in this large and heterogeneous group. The spectrum includes everything from anal stenosis, ventral anus, anal atresia (with and without fistula) and the full spectrum of cloacal malformations. Imaging in these conditions is done through the whole armamentarium of radiologic modalities, with very different imaging strategies seen across the centres where these conditions are managed. In 2017, the European Society of Paediatric Radiology (ESPR) abdominal imaging task force issued recommendations on the imaging algorithm and standards for imaging anorectal malformations. This was followed by further letters and clarifications together with an active multispecialty session on the different imaging modalities for anorectal malformations at the 2018 ESPR meeting in Berlin. Through this paper, the abdominal task force updates its guidelines and recommended imaging algorithm for anorectal malformations.

## Introduction

Anorectal and cloacal malformations are a broad mix of congenital abnormalities related to the distal rectum and anus. There is a male predominance with a prevalence of around 1:5,000 [1] for anorectal malformations (ARMs), whereas cloacal malformations are much more uncommon (1:50,000) and seen almost exclusively in girls. Cloacal malformation represents a common perineal outflow tract of the urinary tract, vagina and rectum.

Although considered the most serious form of ARMs, cloacal malformations represent a further broad spectrum of conditions with multiple associations (specific anomalies and syndromes) with respective imaging and treatment implications.

**Fig. 1:**
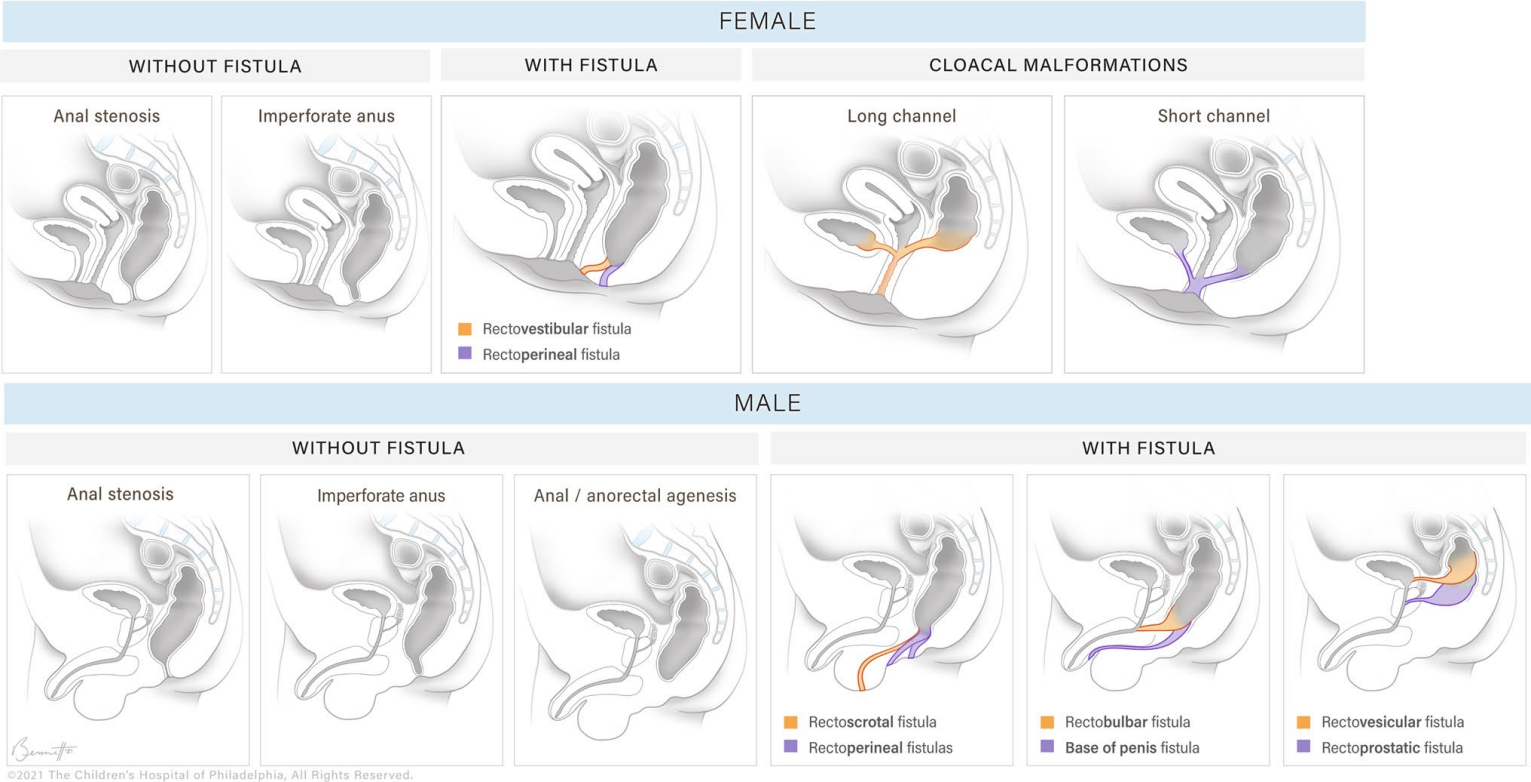
These sagittal illustrations demonstrate the more common anorectal anomalies in males and females (Illustration by Brittany Bennett, MA ©2021 Children's Hospital of Philadelphia. All rights reserved)

Confusion exists between all the forms in this large and heterogeneous group. The spectrum includes everything from anal stenosis, ventral anus, anal atresia (with and without fistula) and the full spectrum of cloacal malformations (Fig. 1).

There is a high incidence of associated congenital anomalies with ARM (Table 1).

**Table 1:** Associations of anorectal malformations (ARMs) (Adapted from Alamo et al. [2])

1a: ARM and associated anomalies	
Cardiovascular Tetralogy of Fallot, septal defects, dextrocardia, aortic coarctation	
Gastrointestinal Oesophageal atresia, small bowel atresias, malrotation and volvulus, Meckel diverticulum, absent colon	
Urogenital Vesicoureteric reflux, dilated pelvicalyceal system, renal agenesis, renal dysplasias, renal ectopia, horseshoe kidney, renal duplication, dilated ureter (megaureter), bladder extrophy, hypospadias, micropenis, duplication of the uterus and vagina, ambiguous genitalia, vulvovaginal atresias	
Spine Sacral agenesis, vertebral anomalies, spina bifida, tethered cord	
Musculoskeletal Hip dysplasia, clubfoot, polydactyly, syndactyly, limb deficiency, Madelung deformity, arthrogryposis	
1b: Multisystem conditions and ARM	
Groups/associations of congenital anomalies The association of vertebral defects, anal atresia, cardiac defects, tracheo-oesophageal fistula, renal anomalies and limb abnormalities (VACTERL association) The association of omphalocele, bladder exstrophy, imperforate anus and spinal defects (OEIS association) The association of Müllerian, renal, cervicothoracic and somite abnormalities (MURCS complex)	
Chromosomal abnormalities Trisomies (13,18,21), Prenatal unidisomy 16, 22q11.2 deletion 13q deletion, heterotaxia	
Syndromes Baller-Gerold, cat-eye, caudal regression, Christian, Currarino, facio-auriculo-vertebral, Feingold, fetal alcohol, FG, Fraser, Ivemark, Johanson-Blizzard, Kabuki, Klippel-Feil, MIDAS (microphthalmia, dermal aplasia and sclerocornea), Okihiro, Smith-Lemli-Opitz, Pallister-Hall, Pallister-Killian, Rieger, Townes-Brock, ulnar-mammary, Walker-Warburg	

Imaging in these conditions is done through the whole armamentarium of radiologic modalities with very different imaging strategies seen across the centres where these conditions are managed.

Correct identification and diagnosis have significant surgical and outcome implications – the management targets being long-term continence and good quality of life.

## Classifications

The accepted classification for ARMs is now the clinically oriented Krickenbeck classification (Table 2) established in 2005 [2, 3]. Considerations that better inform surgical decisions include:

1. The position of the rectal pouch.
2. The presence or absence of any fistula.
3. The type and location of fistula if present.

**Table 2:** Krickenbeck classification of anorectal malformations (ARMs)

Major clinical groups: Anal stenosis ARMs with no fistula Perineal (cutaneous) fistula Rectourethral fistula Prostatic Bulbar Rectovesical fistula Vestibular fistula Cloaca Long channel: more than 3 cm Short channel: less than 3 cm	
Rarer/regional types: Pouch colon atresia/stenosis Rectal atresia/stenosis Rectovaginal fistula H-type fistula Other	

In 2017, the European Society of Paediatric Radiology (ESPR) abdominal imaging task force issued recommendations on the imaging algorithm and standards for imaging ARMs [1]. This was followed by further letters and clarifications [4, 5] together with an active multispecialty session on the different imaging modalities for ARM at the 2018 ESPR meeting in Berlin (ESPR Berlin,19 June 2018, abdomen session III). During this session, it became even more evident that there are clear differing opinions and approaches on the best way to image ARM. Although listed in this classification, cloacal malformations are quite a unique malformation that exhibit differing clinical behaviour based not only on the length of the common channel but also on the urethral length [5].

There have been several scientific publications in the last 24 months targeting these topics and, as such, in consideration of all the above, the ESPR abdominal imaging task force believed it necessary to further update their imaging recommendations.

From the recent literature and targeted sessions, questions that remained relatively unanswered include:

1. What is the role of antenatal imaging in diagnosis?
2. Which exact imaging examinations are recommended before the early surgical decision (a primary perineal approach or abdominal diversion of the bowel with perineal surgery at a secondary stage)?
3. When the colon gets rerouted during early surgical management, what follow-up imaging is recommended before perineal surgery? Which, when and how should such interim imaging be performed?

From a modality perspective:

1. Is perineal ultrasound (US) required at birth? What information can/cannot this examination provide? Are there differences in the information provided (i.e. estimation of rectoperineal distance) between US and radiographs?
2. Is a fluoroscopy/voiding cystourethrogram (VCUG) study required at birth? Can such a test be reliably replaced by US/contrast-enhanced ultrasound (CEUS)/US genitography?
3. Is cross-sectional imaging necessary before definitive surgery? If so, when (which scenarios or conditions)? If required, at what age should these be performed? Can cross-sectional imaging replace other examinations? Is computed tomography (CT) still justified, or can it be replaced by magnetic resonance imaging (MRI)? If CT is still justified, when should it be done?

## What is the role of antenatal imaging?

The bulk of children with ARMs are identified at birth. Recent literature has further emphasised the early diagnosis of the spectrum of diseases suggesting roles for antenatal US and MRI. US has been reported to have a detection level of around 30% [6]. MRI is reported to be superior to US; sensitivity in MRI depends very much on the subtype of the anomaly in question and the MRI technique/protocol used. With the sub-spectrum of cloacal anomalies, sensitivity in prospectively identifying the anomalies is reported to be higher. Antenatal MRI also provides relevant further information [6].

### Assessment of the neonate

The clinical assessment by the neonatologist and experienced paediatric surgeon can, in a significant number of cases, determine the type of ARM and allow a clinical decision to be made (regarding early definitive perineal surgery versus colostomy and later perineal surgery). The distention of the rectum and passage of meconium through any fistula present is dependent on distention of the abdomen and gas reaching the distal bowel. Hence, examination soon after birth may be unreliable. [7]

Imaging is needed for further work-up in cases that are not clear-cut at the bedside. The more local radiologic expertise available, the more imaging can help surgeons better identify the subset of conditions involved, make the correct surgical plan and give the best informed consent to the parents.

### Goal of management

The ultimate goal of the surgical management of such infants is bowel control paired with a good quality of life [8]. Common postoperative issues remain soiling and constipation. Surgical definitive management includes the posterior sagittal anorectoplasty (PSARP) versus the laparoscopic or laparotomic approach (LAARP).

The location of the rectal pouch in relation to the levator ani muscle and the presence and insertion of fistulas guide such surgical decisions [8].

## Postnatal imaging

- *Which imaging examinations are recommended before the early surgical decision?*

### Conventional radiography: initial assessment

#### The invertogram or prone cross table radiograph

The standard initial investigation, which has a historical and practical value is the invertogram; this is no longer very much in use and has been replaced by the prone cross table radiograph. For the reasons explained above, this investigation should not be performed on the first day (to allow bowel gas to reach and distend the more distal aspect of the rectal pouch). Striated distal sphincter muscle may prevent the radiograph from outlining the most distal part of the rectum [9]. If this examination demonstrates a short rectoperineal distance, it supports a decision toward early surgery (a true-positive, false-negative scenario) [7].

Due to associated anomalies, the chest and spine should also be imaged by conventional radiographs during the early assessment.

#### Ultrasound: initial assessment

US plays many parts in the early assessment of ARM.

In line with the identified multiple associated other anomalies:

1. The renal tract and pelvis should be scanned early, as well as the entire abdominal cavity.
2. Sonography of the pelvis should attempt to identify the Müllerian structures (in a girl) and exclude a presacral mass – an important exclusion before any pelvic surgery is performed [5].
3. It is also recommended that the spine be imaged focusing on depiction of a possible tethered cord or any other cord abnormality (particularly in a cloacal malformation).
4. The brain should be scanned in suspected associated syndromic conditions.
5. A detailed US does not need to be done as an emergent investigation but as soon as local expertise becomes available.

There has been a lot of literature in support of early perineal US with multiple options of how this can be performed at an early stage as follows:

1. Perineal US (utilising appropriate high frequency probes) to measure the rectoperineal distance and to search for any fistulas.
2. Where a urinary bladder catheter can be successfully passed – US assessment can be augmented with simple instillation of saline - if not sufficient, CEUS can further help identify fistulas when the expertise is available.
3. In suspected cloacal malformation, US can play a role in assessing the complex pelvic anatomy again augmented by retrograde saline infusion of the cloacal common channel or with intracavitary CEUS.
4. More recent literature reports that:
  - Perineal US and conventional radiographs have similar results when measuring the rectoperineal distance [10]. This is, however, being challenged for a number of reasons, including the effect on such distance from the pressure by the US probe and meconium in the most distal part of the rectal pouch [11].
  - US, as in conventional radiography assessment, should also not be performed on day 1 as it may also incorrectly estimate the distance if performed early [12].
  - Perineal US is better than suprapubic US at detecting fistulas [13].
  - Initial US is better at identifying fistulas than a same-day VCUG [14].
  - An initial VCUG/genitography is, however, better to anatomically localise a fistula if a fistula has indeed been identified [14].

#### Fluoroscopy: initial assessment

In presurgical assessment of ARM (definitive or rerouting), fluoroscopy has been proposed to have two main roles:

1. Initial VCUG (before any surgery) where it has been shown to be better at localising a fistula than US (as outlined above).
2. Initial genitogram in female neonates with suspected cloacal malformation.

### Postnatal imaging

- *What follow-up imaging is recommended before perineal surgery?*

#### Fluoroscopy: post colostomy assessment

Fluoroscopy is traditionally used to assess the distal loop of the colostomy in infants having a planned later perineal procedure. The loopogram aims to following:

1. Identify the rectal pouch in relation to the perineum with an estimate of the rectoperineal distance.
2. Identify and localise any fistulas.
3. Assess the length of the distal colon to determine if there is enough length for rectal pull-through or manipulation if needed.

The above recommendations come with a caveat: The technique is paramount and a high-pressure colostogram should be performed.

Technical points to emphasise include:

1. The relative higher risk of perforation around the stoma due to the use of a balloon to obtain an appropriate seal and to distend the distal loop under pressure. This complication can be quite subtle and should be actively excluded at the end of the study.
2. The importance of positioning the infant appropriately with a true lateral projection while the loop is distended.
3. The appropriate position of a small radiodense marker at the anal dimple so that this can be identified.
4. The appropriate positioning of a ruler at the time of the study for calibration and measuring.
5. Distention of the whole distal loop to reduce the risk of a false-negative exclusion of a fistula.
6. Awareness that this examination comes with a radiation burden.
7. Awareness that poor technique can lead to misdiagnosis and mismanagement with resulting long term consequences [15].

#### Ultrasound: post colostomy assessment

In infants who have undergone an early colostomy, US may help in the following scenarios:

1. Distal colostomy assessment with saline distention through the distal stoma assessing the distal pouch and its immediate relations.
2. CEUS of the distal colostomy. The latter has recently been shown to match the traditional distal loopogram in a small group of patients with ARM without perineal fistula [16].

- *What is the role of cross-sectional imaging before definitive surgery?*

#### Magnetic resonance imaging

This imaging technique is outlined in detail in the initial ESPR abdominal task force recommendations [1]:

1. Initial papers advocating MRI favoured a modified pelvic MRI (with injection of saline through colostograms/fistulograms) under light sedation or general anaesthetic [17, 18]. More recent literature has moved away from the modified approach with promotion of a standard feed and sleep (“feed and wrap”) pelvic MRI without distending the distal colon loop or injecting into the perineal fistulas [6, 14].
2. With either approach, MRI is advocated as a promising one-stop-shop modality, allowing assessment of not only the whole pelvic contents (particularly in females) and fistulas but also confidently excluding presacral masses, evaluating the spine, evaluating the perineal sphincter muscle complex (at least in older patients) and evaluating the renal tracts, in one sitting [8, 17–20].
3. In two recent publications, conventional high-resolution pelvic MRI is reported to have a higher sensitivity at appropriately detecting the anatomy of the rectal pouch (above versus at versus below the pelvic floor) when compared to the traditional distal colostogram (the recognised gold standard being the surgical findings). For MRI, this correct identification is reported at 88–93% vs. 67–77% for fluoroscopy [8, 19].
4. MRI is reported to have a higher inter-reader agreement than the traditional colostogram [8].

#### Computed tomography

Little further literature has been published in the last 10 years, in line with the expansion of advanced US techniques and MRI in the evaluation of these spectrum of conditions. CT, with its taxing radiation dose, has become more challenging to justify for such cases. Although the technique allows some information on the sphincter muscle complex, it is poor in soft-tissue characterization [19, 21]. In complex cloacal malformations, some potential benefit in depicting the anatomy has been reported through combining low-dose CT, VCUG and excretory urography to obtain 3-D imaging of the malformation [21].

If performed (most likely in resource-poor environments where other modalities may be less accessible), then the emphasis on low-dose techniques should be paramount.

### Inferences and controversies

**Fig. 2:**
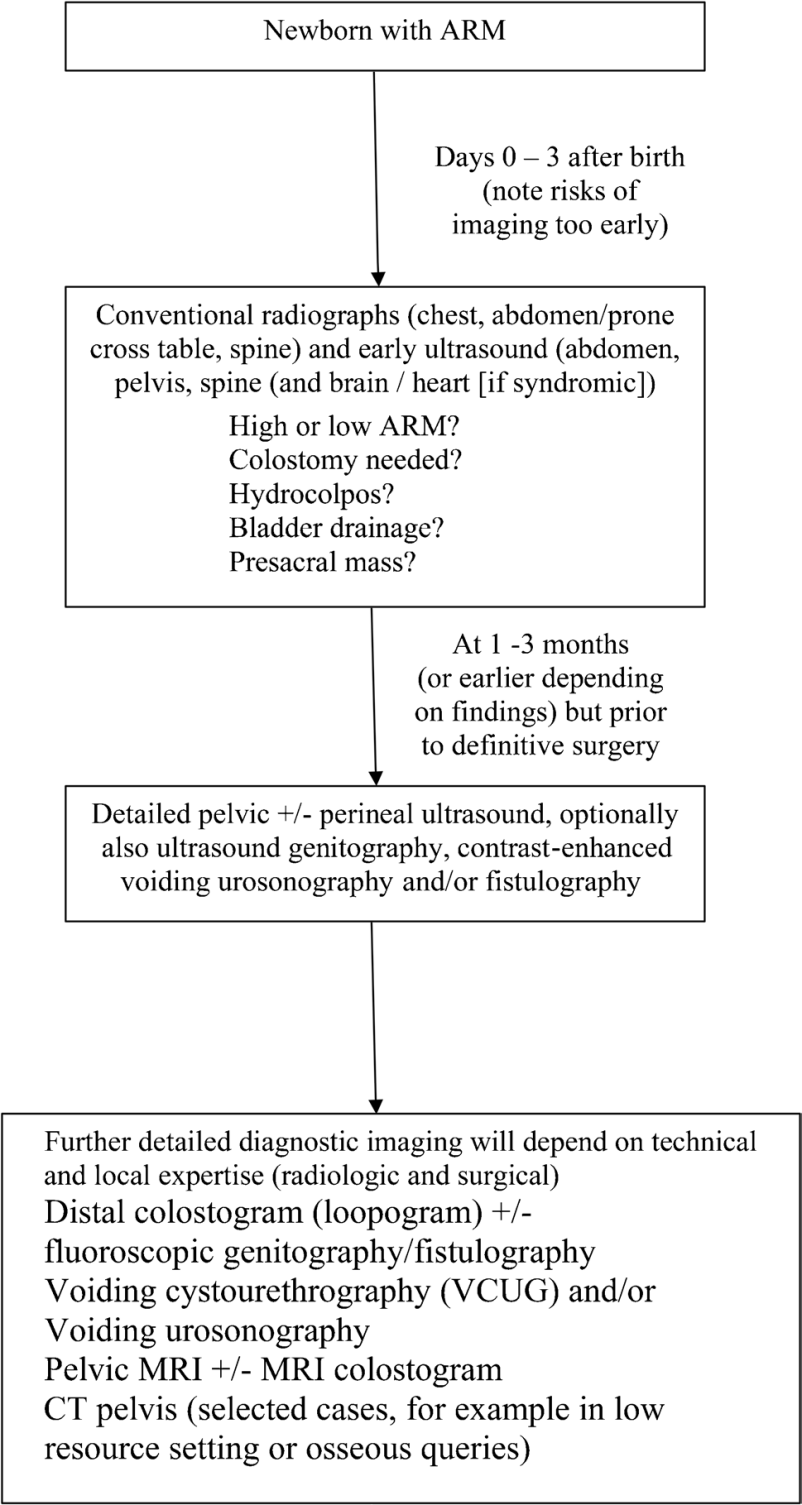
Amended European Society for Paediatric Radiology guidelines for imaging in a neonate with anorectal malformation. *ARM* anorectal malformation, *CT* computed tomography, *MRI* magnetic resonance imaging

From the more recent literature since the ESPR abdominal task force recommendations published in 2017, several inferences can be outlined as follows:

1. Only a few small cohort studies have been published since 2017, again with emphasis on the whole spectrum of different modalities based on the personal/institutional preferences where the research is being undertaken.
2. How much imaging is required at birth before the decision to undergo surgery remains a complex question. Conventional radiography and a detailed early US remain the baseline. The exclusion of an associated presacral mass before any pelvic surgery (through whatever modality is available) must be emphasised. Considering the more recent literature captured in these guidelines, the suggested ESPR guideline algorithm from 2017 [1] has been amended slightly, as per Fig. 2.
3. Paediatric surgeons have different imaging expectations and preferences based on their training and expertise. The spectrum of conditions is very broad and the surgery is very complex. Attendance to the imaging by the paediatric surgeon and working collaboratively as a multidisciplinary team can be very helpful to better understand the complex anatomy, especially for the more dynamic radiologic modalities.
4. A further push toward MRI as a single investigation/adjunct to fluoroscopy is clearly developing with results in more than one study suggesting a better diagnostic correlation with surgical findings when compared to the traditional fluoroscopic assessments. However, there has been no direct correlation with high-level US. Challenges remain when comparing MRI to other modalities and inadequate non-standardised fluoroscopic studies weaken such modality comparisons. Differing MRI protocols and techniques also add to this complexity.
5. Where the radiologic expertise is available, advanced US techniques can be an adjunct or indeed a replacement to neonatal VCUG at birth. However, even research groups strongly advocating for perineal US report that fluoroscopy/VCUG is still required when a fistula is identified to fully characterise it.
6. The role of CT, if any, is harder to justify in the context of all the above developments, except where this is perhaps the only option realistically available to the clinical team, or when associated osseous queries need to be addressed.
7. On a bigger picture, multimodality multi-institutional research studies should be planned with the development of one agreed work-up algorithm being the goal for these infants. The complexity and variability of these conditions, the spectrum of available modalities, some of which are highly dependent on the level of local preference and expertise, might mean that this is a difficult consensus to ever achieve.

## Conclusion

Imaging of ARMs remains challenging and much depends on the local radiologic expertise in the relevant specialised modalities and the local surgical expertise in the management of this broad spectrum of conditions. These new guidelines and updated algorithm better capture the above realities in line with the more relevant recent literature.
